# Extracellular recordings reveal absence of magneto sensitive units in the avian optic tectum

**DOI:** 10.1007/s00359-014-0947-6

**Published:** 2014-10-04

**Authors:** Edgardo Ramírez, Gonzalo Marín, Jorge Mpodozis, Juan-Carlos Letelier

**Affiliations:** Department of Biology, Facultad de Ciencias, Universidad de Chile, Santiago, Chile

**Keywords:** Magnetoreception, Optic tectum, Tectofugal, Avian vision

## Abstract

There is a consensus that birds detect the earth’s magnetic field and use some of its features for orientation and homing purposes. Since the late 1960s, when the first solid behavioral evidence of magnetoreception was obtained, much research has been devoted to describing the ethological aspects of this behavior. The neurophysiological basis of magnetoreception has been much less studied, although a frequently cited 1986 report described a high prevalence (70 %) of magneto-sensitive neurons in the pigeon optic tectum with high signal-to-noise ratios (Semm and Demaine, J Comp Physiol A 159:619–625, 1986). Here, we repeated these neurophysiological experiments using anesthetized as well as awake pigeons and new recording techniques. Our data indicate that magneto-sensitive units do not exist in the avian tectum.

## Introduction

Since the mid–nineteenth century, it has been suggested that birds might use the earth’s magnetic field to migrate. It was not until the second half of the twentieth century, however, that avian magnetoreception began to be systematically tested. Early experiments were simple procedures in which magnets were attached to the heads or wings of homing pigeons (Yeagley and Whitmore 1947), and consequently gave confusing results (Gordon 1948; Orgel and Smith 1954). More elaborate experiments, between 1968 and 1974, demonstrated avian magnetoreception in European robins and pigeons using behavioral procedures coupled with alterations of the earth’s magnetic field. In robins (Wiltschko 1968), magnetoreception was confirmed using a simple variation of the Emlen funnel paradigm as robins’ migratory tendency was affected by presenting caged birds with artificially produced magnetic fields during the migratory season. In pigeons, which are non-migrating birds, the demonstration was based on disrupting homing behavior using magnets (Keeton 1971) or by induced magnetic fields (Walcott and Green 1974).

Since the above experiments, the field of avian magnetoreception has been mostly focused on discovering the behavioral subtleties of avian magnetoreception (Winklhofer 2010) and, after four decades of research, the consensus is that magnetoreception is an integral part of the avian sensory world. Nevertheless, there are many unknowns remaining in this field. One unresolved point is the nature of the transducing mechanisms. Two transducing mechanisms have been invoked: one depends on small clusters of magnetic material (magnetite) found in the upper beak (Winklhofer and Kirschvink 2010; Mouritsen and Hore 2012) or in the lagenae (Wu and Dickman 2011). The other model, known as the chemical hypothesis, exploits a quantum property of the excitation of electrons by green or blue photons (Ritz et al. 2000; Schulten et al. 1978; Rodgers and Hore 2009), and recently, a detailed molecular mechanism, involving the flavin cofactor of the cryptochrome protein, has been advanced following this hypothesis (Solov´yov et al. 2014).

Another unresolved aspect concerns the neural responses and pathways involved in magnetoreception. Due to the apparent complexities of performing neural recordings in awake birds, very few groups have explored the neurophysiological basis of magnetoreception. In the early 1980s, four studies, executed essentially by the same group, systematically searched for magneto-sensitive neural responses in the avian brain. Using extracellular recordings, these studies explored the pineal gland (Semm 1983), the vestibular nuclei, the nucleus of Basal Optic Root (nBOR) (Semm et al. 1984), and the optic tectum (OT) (Semm and Demaine 1986) of pigeons. They also looked for magneto-sensitive neurons in the trigeminal ganglion of the bobolink (*Dolichonyx oryzivorus*) (Semm and Beason 1990a).

The 1986 results concerning the pigeon’s optic tectum were particularly striking as they reported that a large fraction of tectal neurons (70 %) were magneto-sensitive and had a large signal-to-noise ratio (>5). Furthermore, some of the magnetic responses were wavelength dependent, a fact supporting the chemical hypothesis of magnetoreception. Thus, it is not surprising that this study has been steadily cited in several studies as proof of the existence of magneto-sensitive units in the avian brain (Able 1994; Azanza and Del Moral 1994; Ahmad et al. 2007; Brasel et al. 2007; Burger et al. 2010; Buttemer and Chappell 2010; Bischof et al. 2011; Begall et al. 2013; Beason 2005; Beason et al. 1995, 1997; Bingman et al. 1988; Cain et al. 2005; Deutschlander et al. 1999; Edmonds 1992, 1993, 1996; Finney 1995; Fischer et al. 2001; Johnsen and Lohmann 2005; Jorge and Vicente 2006; Keary and Bischof 2012; Kobayashi and Kirschvink 1995; Leucht 1990; Liboff and Jenrow 2000; Lohmann and Lohmann 1993; Lohmann and Johnsen 2000; Mai and Semm 1990; McKay and Persinger 2005; Mehlhorn and Rehkamper 2009; Mouritsen et al. 2004; Muheim et al. 2002; Munro et al. 1997; Nemec et al. 2001, 2005; Niessner et al. 2011; Olcese and Hurlbut 1989; Olcese et al. 1988a, b; Partch and Sancar 2005; Phillips 1996; Phillips and Borland 1992a, b; Phillips and Borland 1994; Phillips et al. 2010; Phillips and Sayeed 1993; Picazo et al. 1993; Ritz et al. 2000, 2002; Rowe 1999; Schneider 1995; Semm and Beason 1990b; Shcherbakov and Winklhofer 1999; Stehle et al. 1988; Taube 1998; Thoss and Bartsch 2003; Thoss et al. 2000, 2002; Tian et al. 2007; Vargas et al. 2006; Walcott et al. 1988; Wallraff and Sinsch 1988; Wiltschko et al. 1993, 1994, 2003, 2004, 2006, 2008, 2010, 2013; Wiltschko and Wiltschko 1995, 2001, 2002, 2005, 2006, 2007; Wu and Dickman 2011; Yano et al. 1996).

Interestingly, after the 1986 description, no new studies concerning the neurophysiology of tectal avian magnetoreception have been published to confirm or to expand their scope. This lack of new studies is rather puzzling as the existence of magneto-sensitive neurons in the avian tectum has important theoretical consequences with respect to our understanding of how different sensory modalities interact to build a singly unitary percept. In effect, the avian optic tectum is already a site of visual and auditory convergence, and the additional existence of a magnetic modality would endow the avian tectofugal pathway with yet another sensory modality. Furthermore, the unambiguous description of magneto-sensitive units would greatly help one to disentangle how the two competing models (magnetite-based or chemical hypothesis) explain magnetoreception. Perhaps a partial explanation of this lack of follow-up neurophysiological experiments could be traced to the small number of laboratories dedicated to performing neurophysiological recordings in birds. Furthermore, researchers working on magneto-sensitive mechanisms using activity-dependent markers, such as *c*-*fos* and ZENK, expressed doubts about Semm and Demaine results as they failed to elicit magnetic-dependent activation in the tectum (Mouritsen and Ritz 2005; Heyers et al. 2010; Mouritsen and Hore 2012; Mouritsen 2013; Zapka et al. 2009; Zapka et al. 2010).

As the original report describing tectal neurons sensitive to magnetic fields has not been replicated or expanded, and neuroanatomical data seems to contradict these findings, we repeated the experiments carried out by Semm and Demaine (1986) by closely following their experimental paradigm, but in addition, using methods and techniques not available in 1986.

## Methods

### Animal preparation

A total of 24 pigeons (*Columba livia*) (21 common pigeons and 3 homing pigeons), with weights between 300 and 480 g, were used. Recording experiments were carried out in anesthetized and awake conditions and homing pigeons were used only under awake conditions. Two anesthesia protocols were used. The urethane protocol (*n* = 5) consisted of a single intramuscular dose of a freshly made 20 % urethane solution in physiological saline of 1 ml per 100 g [this protocol was used in (Semm and Demaine 1986)]. The ketamine–xylazine (kx) protocol (*n* = 12), used in our laboratory in the last decade (Letelier et al. 2004; Marin et al. 2005, 2007, 2012), consisted of an intramuscular dose of 0.35 ml per 330 g of a solution of 1.5 ml of 10 % ketamine plus 0.7 ml of 2 % xylazine, followed by 0.03 ml maintenance doses every 1.5 h. kx anesthesia differs from urethane anesthesia as it allows pigeons to obtain a reliable recovery of physical activity and vigilance after the last maintenance dose of anesthesia. In all acute experiments the cloacal temperature was monitored and a DC-powered electric blanket stabilized body temperature in the 38–40 °C range (Temperature Control Unit from Frederick-Haer Co). Furthermore, the ECG was monitored and displayed, and the heart beat frequencies calculated and displayed. In acute experiments, pigeons were placed in a custom-made brass/plastic stereotaxic frame allowing for presentation of visual and magnetic stimuli as well as easy manipulation of microelectrodes and access to perform a craniotomy above the left optic tectum which, in pigeons, receives retinal axons exclusively from the right eye. The craniotomy enabled visual approximation with microelectrodes to the accessible tectum, with the tectal area corresponding to a 25° solid angle around the pigeon’s optic axis (Letelier et al. 2004).

### Awake pigeons

For experiments under awake conditions (*n* = 6), pigeons underwent, at least 2 days before the first recording session, installation of a head-restraining device consisting of two anchoring screws (size 00- or 1.19-mm outside diameter) on the skull, using dental acrylic, for head movement restriction. The protruding threads were used, in the recording sessions, to fix the head to a modified stereotaxic frame. In the preparation session, a craniotomy over the left accessible tectum was also performed. A small Teflon recording chamber sealed the open space over the tectum and provided mechanical isolation.

### Recordings

Recording experiments were performed using either 1-channel tungsten microelectrodes (from Frederick-Haer Co.), a 3-microelectrode array (1 experiment) or 16-channel silicon probes (from NeuroNexus). One-channel microelectrodes were used in all common pigeons and 1 homing pigeon, and 16-channel probes were used in three homing pigeons. All electrodes were mounted on a Narishige micromanipulator (MMD-4) attached to the stereotaxic apparatus.

### Recorded nuclei

Left and right tecta were recorded under urethane anesthesia; the left tectum was recorded under ketamine and in awake pigeons. Recordings in the tectum were performed at three different depths: superficial (300 μm), intermediate (550 μm), and deep (800 μm) layers. In one occasion, recordings were done in the Isthmi parvocellularis (Ipc) nucleus, an important component of the avian visual attentional circuit (Marin et al. 2007, 2012).

### Sampling

Extracellular signals were amplified (1,000× or 5,000×), filtered (band pass between 5 and 5 kHz) by a Model 3600 16 channels AC-Amplifier (AM-Systems), and sampled at 10 kHz by custom-made software done in Igor (www.wavemetrics.com). Signals were continuously monitored during data acquisition, and analysis was done offline.

### Data analysis

Data analysis consisted of an initial filtering of raw recordings to obtain local field potentials (LFPs) below 300 Hz and spikes above 300 Hz. In the avian tectum, in the layers corresponding to the stratum *griseum et fibrosum superficiale* (Butler and Hodos 2005), neural activity is swamped by the activation of paintbrush axons (coming from the Ipc nucleus) (Marin et al. 2005); thus, it is impossible to obtain pure unitary activity. Thus, in this study, for each recording site, we analyzed LFP signals and multiunitary spiking activity. To quantify magnetic-dependent effects, the LFP amplitude profile and the spike peri-stimulus histogram were correlated with the amplitude and direction of the applied magnetic field. A positive response was taken as a signal three times the rms value of the control experiment.

### Generation of magnetic field

**Fig. 1:**
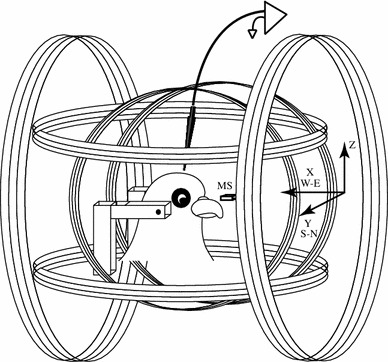
The stimulation setup consisted of three orthogonal Helmholtz coils. The total magnitude of the artificial field was kept similar to the amplitude found in Santiago. Most of the experiments recorded from the left tectum and the right eye was illuminated with LEDs located at 50 cm behind a translucent screen. A 3D magnetic sensor (MS) was located near the pigeon beak. The coils and LEDs were powered by high current 12 V DC batteries. In experiments with awake pigeons the ear bars were not used. The coils left most of the right visual field unobstructed

Special care was devoted to generate and measure the applied magnetic field. Three pairs of single-wrapped Helmholtz coils generated an arbitrary 3D magnetic field with an intensity similar to those found in Santiago, Chile (Latitude = 33°26′16″ South, Longitude = 70°39′1″ West, Altitude = 567 M) of 0.242 Gauss (see model at http://www.ngdc.noaa.gov/geomagmodels/IGRFWMM.jsp). The three-coil pairs were placed orthogonally such that the magnetic field generated by the smaller pair (diameter = 0.60 m and separation = 0.3 m) was along the geographic north–south axis. Another pair (diameter = 1.10 m and separation = 0.55 m) generated an east–west field while the third pair (diameter = 0.80 m and separation = 0.4 m) was aligned to an up–down axis (Fig. 1). This arrangement produced, at the geometric center of the coils, a cube (edge length = 5 cm) where the intensity profile of the magnetic field was within 1 % of a homogenous field (see equations in pages 406 and 407 in Kirschvink 1992 and Ramirez 2011).

The pigeon, attached to the stereotaxic frame, was placed inside the three pairs of Helmholtz coils, with its antero-posterior axis being aligned with the geographic east–west axis (head:east, tail:west), while its right eye was placed at the very center of the three pairs of Helmholtz coils. Thus, in our system, the pigeon head was placed in a region with a uniform magnetic field orientation and magnitude of which could be changed arbitrarily.

To generate magnetic vectors with an arbitrary 3D orientation (or orientation of the artificial magnetic field), the sense and magnitude of the current traversing each pair of Helmholtz coils were computer controlled. A specially made system, based on Igor-Pro (www.wavemetrics.com) and a National Instruments data acquisition card controlled three slow varying currents, ranging from, −1 to 1 Amp. These currents were generated by two 12-V/55-Ah lead batteries. By changing the relative amplitude of the three currents, any 3D orientation was achievable for the artificially produced magnetic field. Every Helmholtz pair was capable of safely producing 1 Gauss field, although in our experiments, the total magnitude of the artificial field was restricted to 0.242 Gauss (earth’s magnetic field intensity at Santiago, Chile). As the magnetic field was under computer control, the control electronics was designed to handle large currents. The batteries’ voltage levels were monitored, and the field was continuously sensed along three orthogonal axes. The end result was a stable and predictable magnetic field.

### Magnetic field measurement

**Fig. 2:**
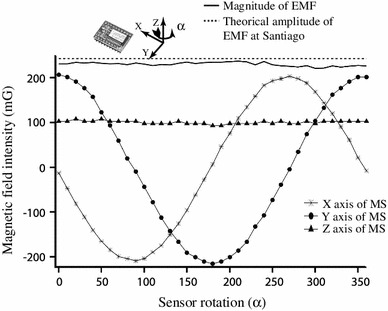
Properties of the 3D magnetic sensor (MS) under a rotation around the *Z* axis. The sensor was placed horizontally and rotated manually around the *Z* axis in 10° steps while recording its three outputs. As expected, the *X* (*asterisk*) and *Y* (*filled circle*) outputs were in quadrature while the *Z* (*filled triangle*) output was almost constant. The total magnitude of the recorded field was 5 % lower (*gray line*) than the theoretical value of the earth’s magnetic field (EMF) at Santiago according to the NOAA-WMM 2010 model (*dotted line*)

The continuous recording of the intensity and direction of the magnetic field produced around the pigeon’s head was achieved by a magneto-triaxial sensor (Honeywell HCM2003) located at 2 cm from the right eye. This sensor, with a sensitivity of 0.0001 Gauss and a maximum range of ∓2 Gauss, was sampled at 12 bits at the same sampling rate as that used for extracellular recordings (10 kHz). The quality of the triaxial sensor was assessed by measuring its *x*, *y*, and *z* outputs, while the sensor was rotated around its vertical (*Z*) axis. The theoretical earth’s magnetic field magnitude at Santiago is 0.242 Gauss, and the sensor measured an average of 0.23 Gauss. The rotation of the sensor did produce the expected signal profiles as the *Z* axis was almost invariant, while the *X* and *Y* axes showed the (in quadrature) sinusoidal variation (Fig. 2).

### Magnetic stimulation

Using Semm and Demaine (1986) rationale, we used slow movements, or scans, of the magnetic field vector as potential stimuli for tectal units. Our stimulation protocol consisted of first canceling out the local Earth’s magnetic field and then generating an artificial magnetic field on a vertical plane, which begins in the vertical axis (pointing upward) and scans 360° of the plane at different speeds: 1, 3, 4, 6, 12, 24, and 48°/s. This procedure was selected because evidence indicates that birds are sensitive to the vertical component of the magnetic field (Beason 2005). Our magnetic scans explored a wide range of speeds. We used not only 1°/s (as Semm and Demaine) but also tested significantly faster scans (12, 24, and 48°/s) in order to elicit magnetic responses. Scans slower than 1°/s were not used. Essentially, we compared neural activity, and either spike data or LFP, between experimental (artificial magnetic field ON) and control (artificial magnetic field OFF) conditions.

### Calibration of magnetic coils and crosstalk measurements

**Fig. 3:**
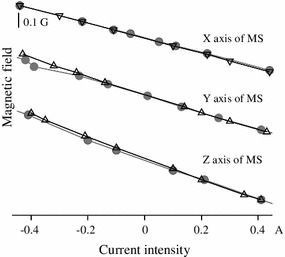
Behavior of Helmholtz coils. The magnetic intensity produced by each Helmholtz coil pair used in isolation (*open triangle*) is a linear function of the current traversing each pair (data for all three pairs). When the three coils are energized concurrently, the deviations are minor (*filled circle*). Thus, the *X*, *Y* and *Z* components of the induced magnetic field are almost orthogonal

An important consideration, besides controlling for the total amplitude of a generated magnetic fields, is to check for the amount of crosstalk existing in our system due to the nonorthogonality of the coils’ frame and other factors. To effectively characterize our stimuli, it is important to measure the degree of crosstalk between the North/South, East–West and Up–Down magnetic fields. To assess how much the physical implementation of our system produced orthogonal magnetic fields at the central location, we generated a single magnetic field in one dimension and measured how much field was detected in the other two orthogonal dimensions. Figure 3 shows the linearity and how much crosstalk, or leakage, existed in our setup. For example, for the *X* field, the intensity of the *X*-axis output of the 3D magnetometer is linear with respect to current when only the *X*-axis coils were energized (open triangle) or when all three coils were energized (filled circle). Similar results hold for the *Y* and *Z* axes.

### Photo-stimulation

The right eye was illuminated using red/blue/green LEDs (Blue:77 Lux, Green:130 Lux, Red:73 Lux, darkness: 5 Lux). The photostimulation protocol consisted of performing control recordings (i.e., in darkness and without magnetic stimulation) followed by recordings done under magnetic stimulation under four conditions: darkness, red light, green light and blue light. The pigeons were given 2 min of photo-stimulation—only before beginning the recordings under magnetic stimulation to ensure that they had time to get accustomed to the new lighting condition.

## Results

**Fig. 4:**
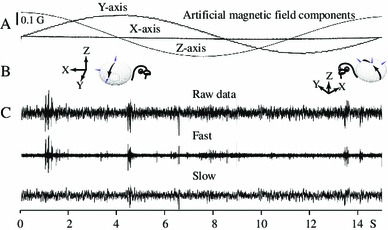
Neural data under magnetic stimulation in an awake pigeon under blue light. The applied magnetic field starts vertically aligned with the *Z* axis, and it is rotated, counterclockwise, in the (*Z*, *Y*) plane in 15 s. **a** The *X*, *Y*, *Z* outputs of the magnetic sensor show the expected sinusoidal variations around the *Z* and *Y* axes, **b** diagram showing the relative position of the magnetic field reference frame with respect to the pigeon’s head, the small sphere shows the movement of the magnetic vector with respect to the pigeon’s head (*left inset* = scan begin; *right insert* = scan end). **c** Neural signals (raw, fast and slow) obtained during magnetic stimulation. Bursty activity occurs at 1, 4.5 and 13.5 s, but it is not related to the applied magnetic field

**Fig. 5:**
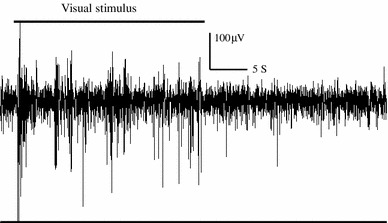
Neural data under visual stimulation in awake pigeon. Tectal activity (raw signal) elicited by the slow movement of a 1° spot traversing a tectal receptive field. The stimulus was manually moved during the first 25 s and trigger strong and clear responses. When the movement ceased, neural activity disappeared. This response shows how strongly tectal units respond to visual motion

We recorded data from 24 pigeons from 91 sites with clear visually driven activity. These sites, located in the accessible tectum, were found above tectal layer 13 and correspond to the stratum *griseum et fibrosum superficiale.* All sites exhibited strong and clear sensitivity to motion; their receptive fields were located 25° from the optic axis and had the typical bursty response found in the avian tectum. Our first qualitative observations show that, in the pigeon’s optic tectum, magneto-sensitive units, contrary to the Semm and Demaine’s (1986) description, are essentially absent. Figure 4 shows a typical pattern of raw neural activity when an artificial magnetic field is rotated (counterclockwise) around the (*Y*, *Z*) plane (at a scanning speed of 24°/s) of an awake pigeon. Normal, spontaneous tectal bursty activity appears (see bursts at 1, 4.5, and 13.5 s), but it is not possible to demonstrate a clear modulation of neural activity by the applied magnetic field. For the purpose of comparison, we show the robust tectal activity normally triggered by visual stimulation (Fig. 5).

**Fig. 6:**
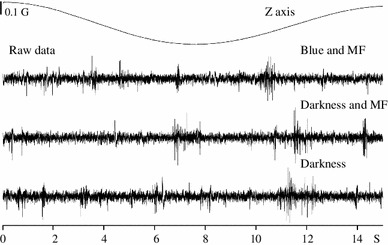
Neural data under different stimulation conditions in awake pigeon. Raw activity traces obtained under three experimental conditions; blue illumination with artificial magnetic field (*upper trace*), darkness with artificial magnetic field (*middle trace*) and darkness with no artificial magnetic field (*lower trace*). In all three conditions, tectal bursts are visible

Perhaps the lack of strong magnetic modulation could be traced to an improper *visual* costimulation (Fig. 6), but the patterns of neural activity under a variety of background lighting (i.e., blue vs darkness) were similar. Tectal activity was equally un-modulated if the applied magnetic vector moved in the (*X*, *Z*) plane (data not shown).

**Fig. 7:**
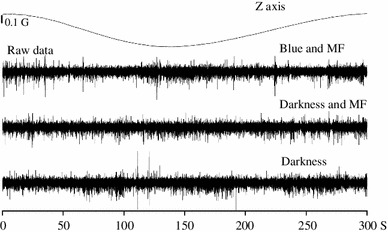
Neural data under magnetic stimulation in an awake pigeon under blue light, at 1.2°/s. Tectal raw activity elicited by the slow scan (300 s) of the artificial magnetic field under three stimulation conditions. No modulation of neural activity by the magnetic field was apparent (compare with Fig. 6)

Alternatively, the lack of modulation could be caused by a *fast* magnetic scanning velocity (Semm and Demaine used 1.4°/s). Figure 7 shows the neural activity when the magnetic scan velocity was 1.2°/s. At least, in this level of standard description, it was not possible to detect any obvious change in LFP activity or multinitary spikes by the applied magnetic field (see Fig. 1a, b of Semm and Demaine (1986) where a magnetic response seems to have a signal-to-noise ratio between 5 and 10). With the techniques based either on LFP amplitude or on spike histograms, we only found one site (in 91)—an urethane anesthetized pigeon—that increased its LFP activity with the application of the magnetic field.

To complement our analysis, and with the hope to find more subtler correlations, we did a deeper analysis in a subset of recording sites focusing on revealing modulatory effects either for spike or LFP data.

### Spike analysis

**Fig. 8:**
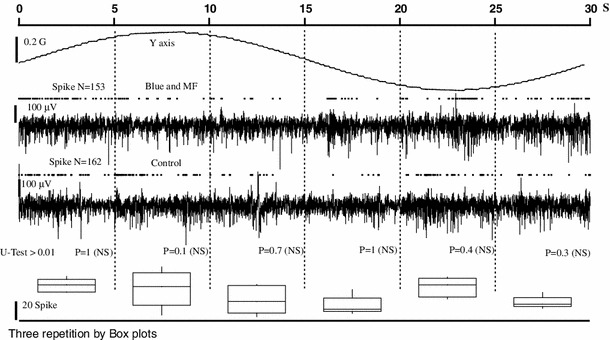
Segment method to assess responsivity of tectal units in a magnetic field (spike signal). The two neural signals represent the multiunit activity of tectal neurons in control (*third trace*) and in the presence of a magnetic field and blue light (*second trace*) for the indicated magnetic field (*first trace*)—above neural traces spikes are represented by *dots*. A first observation reveals that the raw activity (measured in terms of total number of detected spikes per stimulation cycle) is not different between the two conditions (153 vs 162 spikes). As these experiments were done in an awake pigeons, a spontaneous and variable discharge was observed. To assess a possible magnetic effect, the complete run was divided in six 5 s segments. The total number of spikes in each segment in both conditions was compared with the *U* Mann–Whitney test (aggregating the three repetitions done for every condition). The *U*-statistics, given below the control trace, show that no difference could be detected for experimental and control conditions [the *p*-statistics were larger that 0.01 thus differences were not significant (NS)]. Furthermore, to show the intrinsic variability of discharge rate, we considered together the spike data from control and experimental conditions, and we represented these sets (6 numbers) by their *boxplots* (lower panel). It is immediately apparent how variable is the discharge rate in awake pigeons (compare *boxplots* from the second and fourth segments). In all nine experiments we could not detect a modulation of the discharge rate

For a subset of recording sites (*n* = 6), we did a quantitative analysis trying to reveal subtle modulatory effects triggered by magnetic stimulation. In these sites, we analyzed multiunitary data by grouping together spikes without attempting to perform spike sorting. This type of grouping is normally done in the tectum, as tectal activity between layers 1 and 10 is swamped by a large, extremely variable, response due to paintbrush axons (Marin et al. 2007). A first observation, confirming previous descriptions, is that tectal neural activity, when not visually stimulated, shows a low level of spontaneous (i.e., nonvisual) activity. Because the basal discharge rate is so low, and magnetic effects (if present) are not very potent, the simple comparison of spike rates before/after stimulus presentation failed to discover spiking rate modulations. Figure 8 exemplifies this analysis for a 30-s magnetic scan (first trace) equivalent to 12°/s. The two neural signals represent tectal multiunitary activity in the absence (third trace) and the presence of a magnetic field and blue light (second trace). Above each neural trace, spikes are represented by dots. As this experiment was done in an awake pigeon, a spontaneous and variable discharge was observed. Raw activity (measured in terms of total number of detected spikes per stimulation cycle) is not significantly different between experimental and control conditions (153 vs 162 spikes).

The assessment of a possible magnetic effect was done by segmenting each experimental or control scan in six 5 s segments and comparing the total number of spikes in each segment in both conditions with the Mann–Whitney *U* test (aggregating the three repetitions done for every condition). The *U*-statistics, given below the control trace, show no differences between experimental and control conditions [the *p*-statistics were larger than 0.01; thus, differences were not significant (NS)]. Furthermore, to show the intrinsic variability of discharge rate, we considered together the spike data from control and experimental conditions, and we represented these sets (6 numbers) by their boxplots (lower panel). It is immediately apparent how variable the discharge rate in awake pigeons is; in all the six sites, we could not detect a modulation of the discharge rate. Table 1 summarizes these data (obtained in awake and anesthetized animals). First, the segment by segment comparison between experimental conditions was not significant (3rd column, Kruskal–Wallis test) and second, the overall comparison between any experimental segment against all the control segments was also not significant (4th…9th columns, Kruskal–Wallis test). Taken together, these data imply that, if some magnetic effect associated to a particular phase of scan exists, then it must be a weak effect.

**Table 1:** Spike rate variability

	Magnetic scan velocity (°/s)	Spike rate variability (Kruskal–Wallis statistic, *p* value)						
		Intra-experimental runs	Experimental (segment number) vs control (overall data)					
		All segment	1st segment	2nd segment	3rd segment	4th segment	5th segment	6th segment
Awake	12	0.07	0.19	0.13	0.36	0.32	0.41	0.21
	6	0.13	0.012	0.72	0.29	0.23	0.31	0.25
	3	0.03	0.9	0.9	0.9	0.58	0.58	0.05
Anesthetized (ketamine)	24	0.13	0.79	0.38	0.11	0.43	0.88	0.25
	12	0.03	0.11	0.72	0.4	0.4	0.29	0.17
	6	0.02	0.65	0.62	0.12	0.11	0.09	0.17

To assess the possible existence of small magnetic-dependent changes on spike rate the Kruskal–Wallis statistic was used. Each run (experimental or control) was divided in six equal duration segments and the Kruskal–Wallis statistic, with respect to the total number of spikes in each segment, was need to assess variability. Column 3 (Intra-experimental runs) measured variability, in experimental runs, by comparing the activity in each segment against all the others from the same experiment for three repetitions. As the *p* value is above 0.01 this indicates that all segments had essentially the same spike rate and same variability. In columns 4–9, we compared spike rate in each experimental segment against all the control segments, again no discernable effect is apparent. The variability was the same for awake or anesthetized animals and independent of magnetic scan velocity

### Local field potential analysis

**Fig. 9:**
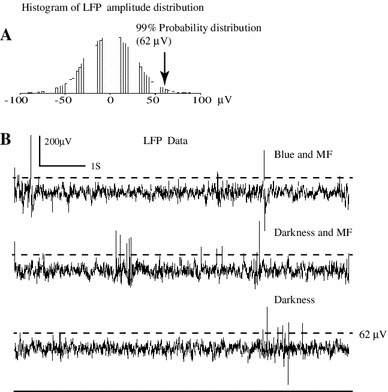
Method to assess responsivity of tectal units to magnetic field using LFP amplitudes. The slow signal from the control situation was used to build a histogram of amplitudes (**a**). The *x*-coordinate corresponding to 99 % was calculated (*arrow*). This value was used to calculate the amount of time that neural signals in experimental conditions were above this value. If the amount of time that the signal was above this limit was higher than 2 % we counted it as a positive event (**b**)

For a subset of nine experiments we did a quantitative analysis of LFP activity between control and experimental runs. In the case of control runs (no external magnetic field) in the dark, we built the distribution of LFP amplitudes. From this distribution we obtained the value corresponding to the upper 1 % (Fig. 9a), and we measured the total time (*t*_d_) when the LFP signal was above this level. Next, we built the same distribution for the experimental condition, and we measured the amount of time when LFP activity was above this critical value. We considered a positive response any run where the LFP signal was above that threshold for a time superior to 2*t_d_. Again no positive effect was found.

**Fig. 10:**
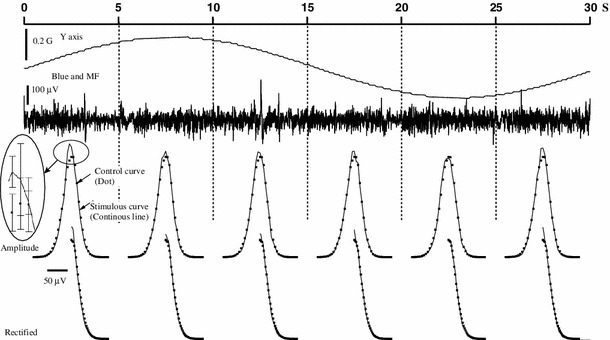
Segment method to assess responsivity of tectal units to magnetic field (LFP signal). A tectal unit was stimulated by varying the vertical component of the magnetic field in 30 s for a complete 360° excursion (*first row*). To assess the existence of possible magnetic responses the 30 s segment was subdivided in six 5-s segments. For each segment we calculate, for control (no magnetic field, trace not shown) and stimulated conditions (magnetic field + blue light, *second row*), the histogram of amplitude values (*third row*) or the histogram for rectified amplitudes (*fourth row*) of the LFP signal. No differences were detected for direct or rectified amplitudes. The empirical distributions were similar for control (*dot*) or for stimulated (*continuous curve*) conditions (*third* and *fourth rows*). The small *inset*, *third row*, shows the standard deviations in both cases, and when they are taken into account, no significant differences are detected between control and experimental conditions. Thus, the magnetic field does not stimulate tectal neurons as it does not influence the amplitude of LFP signals (either direct or rectified) in specific phases of the magnetic field

As magnetic responses could be *specific for certain phases* of the magnetic field (i.e., magnetic vector pointing down), our previous analysis is not adequate as it subsumed all data in a single set. Thus, in a subset of nine experiments (encompassing runs at 15, 30, 60, and 120 s in awake and anesthetized pigeons), we divided each run into six segments. For each segment, we obtained the distribution of direct and rectified (RMS) LFP amplitudes for control and experimental conditions as well as their standard deviations (Fig. 10). In all the nine units the distributions of amplitudes (or rectified LFP) were similar between control and experiments. This detailed analysis failed to reveal any differences in the LFP responses for awake/anesthetized pigeons or for slow/fast presentations of the magnetic stimuli in the different phases of the application of magnetic field.

Table 2 shows a summary of the data and conditions used in this study. Of this set, only one cell did exhibit an increase in LFP activity with a change in the magnetic field. We interpret this single result as the unavoidable outlier that any statistical procedure will produce, especially when the recording intervals are long and the animal is under deep urethane anesthesia, a condition known to trigger irregular spiking.

**Table 2:** Summary of neural data

Pigeon type	Anesthesia	Recording site	Electrode type	Units	Magneto-sensitive unit
4 Normal	Urethane	Left Tectum	1-Channel	4	1
1 Normal	Urethane	Right Tectum	1-Channel	1	0
12 Normal	Ket/Xil	Left Tectum	1-Channel	12	0
3 Normal	Awake	Left Tectum	1-Channel	7	0
1 Normal	Awake	Right IPC	1-Channel	1	0
1 Homing	Awake	Left Tectum	1-Channel	6	0
2 Homing	Awake	Left Tectum	16-Channel	60	0
24 Pigeons	–	–	–	91	1

Summary of 91 recording sites, from 24 pigeons under many different conditions (type of anesthesia, pigeon type, recorded nuclei). Because a systematic exploration was time consuming (A complete scan at 4°/s took almost 1 h. When all the speeds and conditions were considered, it took between 3 and 4 h to complete a data gathering cycle.), it was not possible to test all units under all scanning conditions

## Discussion

Although Semm and Demaine’s (1986) original report has been steadily and extensively cited as proof that magneto-sensitive units exist in the avian visual system, we failed to replicate their findings in the optic tectum where they found up to 70 % of magneto-sensitive responses.

In general negative results are not reported—the lack of evidence could have many subtle and uninteresting causes—but, in this particular case, we think that our negative result must be disseminated. In effect, as we understood the many causes that could hinder the discovery of magneto-sensitive units, we devoted great effort to circumvent them. For example, instead of using a single anesthetic (urethane) we also used the ketamine–xylazine mixture as well as performing experiments in awake pigeons. Besides recording from common pigeons, we also used homing pigeons tested in short successful homing flights. Thus, our failure to detect magneto-sensitive units cannot be traced to the type of anesthetics used or the type of pigeons employed.

The magnetic field was continuously measured and recorded in terms of its direction and magnitude to assure a correct application of the magnetic stimulus. Instead of using an inclinometer [see Methods section of Semm and Demaine (1986)], we sensed the magnetic field with a triaxial sensor around the pigeon head with an appreciable level of accuracy. This continuous monitoring was necessary as the electronics controlling the current in each Helmholtz pair could fail; thus, our online checking of the 3D components of the applied magnetic field was more complete than the systems available in 1986. The 3D sensor was an essential element, as in some experiments, the applied magnetic field disappeared due to electronics or software factors and the reporting system allowed us to apply immediate corrective actions. Thus, we can assert that during all our recordings, the artificial magnetic field was turned on, and thus, our negative results are not due to a lack of properly configured magnetic stimuli.

The type of magnetic stimulation was also expanded from the sweeps between +62° and −62° in the vertical orientation in about 90 s used in 1986. We did similar scans, but between −90 and 90 at different speeds (from 1 to 48°/s). We also did magnetic scans in different planes [as in the (*X*, *Z*) plane]. In some experiments, we even mimicked the magnetic field variations produced at the level of the eye by peculiar avian saccadic oscillations by (Pettigrew et al. 1990; Wallman and Letelier 1993). As avian saccades are composed of 6–12 cycles of a 15°, slightly underdamped, 30-Hz sinusoidal rotation around the optic axis, we modulated the magnetic field (by performing 10 cycles of 30-Hz oscillations with a 15° amplitude) in order to mimic the magnetic variations produced by saccades. Of course, the combinations of different magnetic stimuli (amplitudes, frequencies, 3D trajectories) are infinite, and we have explored only a small subset of stimulus space. A logical exploration of 3D magnetic stimuli will demand a better understanding of the eco-physiology of magnetoreception in order to repeat, in the magnetic domain, the approach pioneered by Lettvin and coworkers in 1959 who used, instead of points of light, stimuli mimicking small insects in their seminal work about vision in frogs (Lettvin et al. 1959).

Also we expanded our recordings to the Ipc nucleus that contains an essential part of an attentional mechanism that seems to be an important component of the architecture of the tectofugal pathway. Ipc recordings failed to uncover any modulation by magnetic fields.

The large discrepancy between the Semm and Demaine report and this study is rather puzzling and not easy to explain. One possibility could be due to the high sensibility of avian tectal neurons concerning motion detection. First, it is necessary to consider a very common approach used in vision research when searching for visual units. In effect, it is common practice that, before attempting a quantitative description of the receptive field of a given visual unit, its response is qualitatively assessed by a combination of manually held stimuli (moving dots of lights, small circles, moving gratings). After the unit has passed some easy qualitative tests, more quantitative approaches are used (moving bars or sinusoidal gratings at different speeds). In the Semm and Demaine report, a similar approach was used as they stated that, to discover magneto-sensitive units, they moved horseshoe magnets in front of the pigeon’s head. The rationale for this qualitative approach was that these magnets (with a field intensity hundreds of times the earth magnetic field) provided a strong magnetic stimulus, and thereby a much faster method to stimulate magneto-sensitive units than the time-consuming procedure based on the magnetic scan using Helmholtz coils. Semm and Demaine must have been aware of the motion detection capabilities of the avian tectum, but not of the rather low detection threshold for motion in the avian tectum. Thus, we speculate that perhaps the moving magnet did have a small visual signature (like a low contrast shadow) that triggered visual responses. Following Semm and Demaine, we also performed qualitative explorations using portable switchable magnets (with a field intensity of 400 Gauss at 5 cm) and never obtained any magnetic modulation. Instead of concocting further explanations about the true nature of this discrepancy, we think that the field of the neurophysiology of avian magnetoreception is a good example of the current problem of reproducibility in science. This problem, recently underlined in the scientific literature (Nature 2013; McNutt 2014; Johnson 2013), emphasizes the necessity of implanting new standards when describing experimental methods and statistical procedures.

**Fig. 11:**
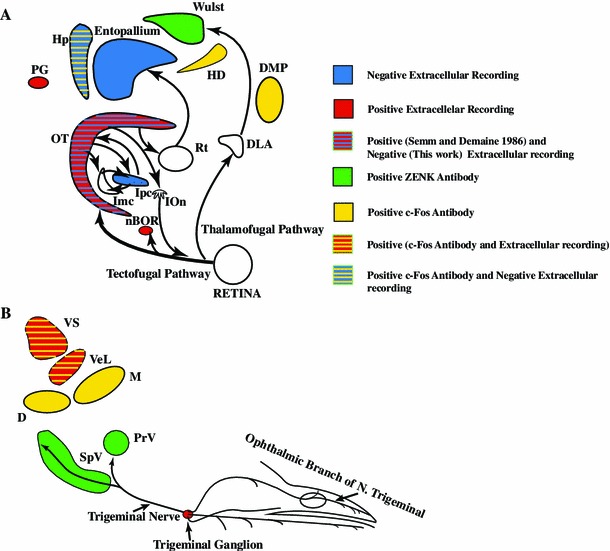
Summary of neurophysiology of avian magnetoreception research. The search for the neural basis of magnetoreception used two different tools: extracellular recordings and activity markers (*c*-*fos*, ZENK). Positive electrophysiological results have been reported in: the pineal gland (PG) (Semm 1983), the trigeminal ganglion (Semm and Beason 1990a, b), the superior (VS) and lateral (VeL) vestibular nuclei (Wu and Dickman 2012), the nucleus of the basal optic root (nBOR) (Semm and Demaine 1986) and the optic tectum (Semm and Demaine 1986). Negative electrophysiological results have been reported in the optic tectum, the nucleus isthmi pars parvocellularis (Ipc), the entopallium and the hippocampus (Hp) (data from Rose 2005 and Ramirez 2011). Positive evidence derived from activity markers has been found in the Wulst, and subdivisions of the hyperpallium (HD, DMP) (Mouritsen et al. 2005; Heyers et al. 2007, 2010; Mouritsen and Hore 2012; Mouritsen 2013). Also subdivisions of the trigeminal nucleus labeling (PrV: Principal sensory nucleus of the trigeminal nerve, SpV: Spinal trigeminal nucleus) (data from Heyers et al. 2010; Wu and Dickman 2011). Some important nuclei, because of their physiology and position in the visual pathways, have not been explored: the nucleus rotundus (Rt), the nucleus of the dorsal thalamus (DLA) and the isthmo optic nucleus (IOn)

Thus, combining the negative results presented here and the equally negative results collected by Rose (2005) who searched for magneto-sensitive units in the pigeon’s entopallium, we have to consider the possibility that extracellular responses sensitive to the earth magnetic field are not as ubiquitous as originally reported by Semm and Demaine. As an extra supporting evidence for this conclusion, we must also consider ZENK studies where no magnetic activation of tectal neurons was elicited although they found magnetic responses at the Cluster N, a nucleus of the retino-thalamo pathway (Mouritsen et al. 2005; Mouritsen and Ritz 2005; Heyers et al. 2007, 2010; Mouritsen and Hore 2012; Mouritsen 2013) (Fig. 11).

Considering all the evidences collected for the avian retino–tecto fugal pathway (Semm and Demaine 1986; Rose 2005; Ramirez 2011; Heyers et al. 2010), we must conclude that the existence of magneto-sensitive units in the avian tectum should not be considered as an established fact.

Our negative results should not be construed as a refutation of avian magnetoreception, or that the retino-tecto fugal pathway is not involved in magnetoreception. In effect, perhaps subtle manipulations of the magnetic stimuli, not reported by Semm and Demaine and considered trivial by them, are necessary to elicit magneto-sensitive responses. Thus, to clarify this long-standing question, recordings with a new level of sensitivity must be carried out in the different nuclei of the different visual pathways. In particular, attention must be paid to the growing evidence, obtained mostly from the c-fos/ZENK studies, pointing to the roles of the retino–thalamic pathway (Heyers et al. 2007; Mouritsen and Hore 2012), the vestibular complex (Wu and Dickman 2011, 2012), and the trigeminal system (Heyers et al. 2010) (Fig. 11). This last system is particularly puzzling as new data show that the iron clusters supposed to be the magnetosensing elements are located in macrophages and not in neurons (Treiber et al. 2012). Also, new evidence—c-fos staining and electrophysiological recordings in the vestibular nuclei (Wu and Dickman 2011, 2012)—detected 16 % of units as being magnetosensitive. However, their stimulation used much faster scans (100°/s) and higher field amplitudes (0.5–1.5 Gauss, corresponding to 1×–3× the normal field intensity at the recording site). These results, if confirmed, open up to yet another possibility for magnetoreceptive mechanisms.

## References

[CR1] Able KP (1994). Magnetic orientation and magnetoreception in birds. Prog Neurobiol.

[CR2] Ahmad M, Galland P, Ritz T, Wiltschko R, Wiltschko W (2007). Magnetic intensity affects cryptochrome-dependent responses in *Arabidopsis thaliana*. Planta.

[CR3] Azanza MJ, Del Moral A (1994). Cell-membrane biochemistry and neurobiological approach to biomagnetism. Prog Neurobiol.

[CR4] Beason RC (2005). Mechanisms of magnetic orientation in birds. Integr Comp Biol.

[CR5] Beason RC, Dussourd N, Deutschlander ME (1995). Behavioral evidence for the use of magnetic material in magnetoreception by migratory bird. J Exp Biol.

[CR6] Beason RC, Wiltschko R, Wiltschko W (1997). Pigeon homing: effects of magnetic pulses on initial orientation. Auk.

[CR7] Begall S, Malkemper EP, Červený J, Němec P, Burda H (2013). Magnetic alignment in mammals and other animals. Mamm Biol.

[CR8] Bingman VP, Ioale P, Casini G, Bagnoli P (1988). Hippocampal ablated homing pigeons show a persistent impairment in the time taken to return home. J Comp Physiol A.

[CR9] Bischof HJ, Niessner C, Peichl L, Wiltschko R, Wiltschko W (2011) Avian UV/violet cones as magnetoreceptors. Commun Integr Biol 4(6):713–716 10.4161/cib.17338 PMC3306339 22446535

[CR10] Brasel JM, Collier AC, Pritsos CA (2007). Differential toxic effects of carbofuran and diazinon on time of flight in pigeons (*Columba livia*): potential for pesticide effects on migration. Toxicol Appl Pharmacol.

[CR11] Burger T, Lucova M, Moritz RE, Oelschlager HHA, Druga R, Wiltschko W, Wiltschko R, Nemec P (2010). Changing and shielded magnetic fields suppress c-Fos expression in the navigation circuit: input from the magnetosensory system contributes to the internal representation of space in a subterranean rodent. J R Soc Interface.

[CR12] Butler AB, Hodos W (2005). Comparative vertebrate neuroanatomy: evolution and adaptation.

[CR13] Buttemer WA, Chappell MA (2010). Ecological and environmental physiology of birds.

[CR14] Cain SD, Boles LC, Wang JH, Lohmann KJ (2005). Magnetic orientation and navigation in marine turtles, lobsters, and molluscs: concepts and conundrums. Integr Comp Biol.

[CR15] Deutschlander ME, Phillips JB, Borland SC (1999). The case for light-dependent magnetic orientation in animals. J Exp Biol.

[CR16] Edmonds DT (1992). A magnetite null detector as the migrating birds compass. Proc R Soc Lond Biol.

[CR17] Edmonds DT (1993). Larmor precession as a mechanism for the detection of static and alternating magnetic-fields. Bioelectrochem Bioenerg.

[CR18] Edmonds DT (1996). Sensitive optically detected magnetic compass for animals. Proc R Soc Lond Biol.

[CR19] Finney B (1995). A role for magnetoreception in human navigation. Curr Anthropol.

[CR20] Fischer JH, Freake MJ, Borland SC, Phillips JB (2001). Evidence for the use of magnetic map information by an amphibian. Anim Behav.

[CR21] Gordon DA (1948). Sensitivity of the homing pigeon to the magnetic field of the earth. Science.

[CR22] Heyers D, Manns M, Luksch H, Güntürkün O, Mouritsen H (2007). A visual pathway links brain structures active during magnetic compass orientation in migratory birds. PLoS One.

[CR23] Heyers D, Zapka M, Hoffmeister M, Wild JM, Mouritsen H (2010). Magnetic field changes activate the trigeminal brainstem complex in a migratory bird. PNAS.

[CR24] Johnsen S, Lohmann KJ (2005). The physics and neurobiology of magnetoreception. Nat Rev Neurosci.

[CR25] Johnson VE (2013). Revised standars for statistical evidence. PNAS.

[CR26] Jorge PE, Vicente L (2006). Light-dependent information: influence of loft conditions on young pigeon’s navigational system. J Ornithol.

[CR27] Keary N, Bischof HJ (2012). Activation changes in zebra finch (*Taeniopygia guttata*) brain areas evoked by alterations of the earth magnetic field. PLoS One.

[CR28] Keeton WT (1971). Magnetic interfere with pigeon homing. PNAS.

[CR29] Kirschvink JL (1992). Uniform magnetic fields and double-wrapped coil systems: improved techniques for the design of bioelectromagnetic experiments. Bioelectromagn.

[CR30] Kobayashi A, Kirschvink JL (1995) Magnetoreception and electromagnetic field effects: sensory perception of the geomagnetic field in animals and humans. In: Electromagnetic fields: biological interactions and mechanisms. Adv Chem Ser (No. 250), pp 367–394

[CR31] Letelier JC, Marin G, Sentis E, Tenreiro A, Fredes F, Mpodozis J (2004). The mapping of the visual field onto the dorso-lateral tectum of the pigeon (*Columba livia*) and its relations with retinal specializations. J Neurosci Methods.

[CR32] Lettvin JY, Maturana HR, McCulloch WS, Pitts W (1959). What the frog’s eye tells the frog’s brain. Proc IRE.

[CR33] Leucht T (1990). Interactions of light and gravity reception with magnetic-fields in *Xenopus*-*laevis*. J Exp Biol.

[CR34] Liboff AR, Jenrow KA (2000). New model for the avian magnetic compass. Bioelectromagnetics.

[CR35] Lohmann KJ, Johnsen S (2000). The neurobiology of magnetoreception in vertebrate animals. Trends Neurosci.

[CR36] Lohmann KJ, Lohmann CMF (1993). A light-independent magnetic compass in the leatherback sea-turtle. Biol Bull.

[CR37] Mai JK, Semm P (1990). Pattern of brain glucose-utilization following magnetic stimulation. J Hirnforsch.

[CR38] Marin G, Mpodozis J, Sentis E, Ossandón T, Letelier JC (2005). Oscillatory bursts in the optic tectum of birds represent re-entrant signals from the nucleus isthmi pars parvocellularis. J Neurosci.

[CR39] Marin G, Salas C, Sentis E, Rojas X, Letelier JC, Mpodozis J (2007). A cholinegic gating mechanism controlled by competitve interactions in the optic tectum of the pigeon. J Neurosci.

[CR40] Marin G, Duran E, Morales C, Gonzalez-Cabrera C, Sentis E, Mpodozis J, Letelier JC (2012). Attentional capture? synchronized feedback signals from the isthmi boost retinal signals to higher visual areas. J Neurosci.

[CR41] McKay BE, Persinger MA (2005). Complex magnetic fields enable static magnetic field cue use for rats in radial maze tasks. Int J Neurosci.

[CR42] McNutt M (2014). Reproducibility. Science.

[CR43] Mehlhorn J, Rehkamper G (2009). Neurobiology of the homing pigeon-a review. Naturwissenschaften.

[CR44] Mouritsen H (2013) The magnetic senses. In: Galizia CG, Lledo P-M (eds) Neurosciences-from molecule to behavior: a university textbook. Springer-Verlag, Berlin, Heilderberg, pp 427–443

[CR45] Mouritsen H, Hore PJ (2012). The magnetic retina: light-dependent and trigeminal magnetoreception in migratory birds. Curr Opin Neurobiol.

[CR46] Mouritsen H, Ritz T (2005). Magnetoreception and its use in bird navigation. Curr Opin Neurobiol.

[CR47] Mouritsen H, Janssen-Bienhold U, Liedvogel M, Feenders G, Stalleicken J, Dirks P, Weiler R (2004). Cryptochromes and neuronal-activity markers colocalize in the retina of migratory birds during magnetic orientation. PNAS.

[CR48] Mouritsen H, Feenders G, Liedvogel M, Wada K, Jarvis ED (2005). Night-vision brain area in migratory songbirds. PNAS.

[CR49] Muheim R, Backman J, Akesson S (2002). Magnetic compass orientation in european robins is dependent on both wavelength and intensity of light. J Exp Biol.

[CR50] Munro U, Munro JA, Phillips JB, Wiltschko W (1997). Effect of wavelength of light and pulse magnetisation on different magnetoreception systems in a migratory bird. Aust J Zool.

[CR51] Nature (2013). Reducing our irreproducibility (*Editorial*). Nature.

[CR52] Nemec P, Altmann J, Marhold S, Burda H, Oelschlager HHA (2001). Neuroanatomy of magnetoreception: the superior colliculus involved in magnetic orientation in a mammal. Science.

[CR53] Nemec P, Burda H, Oelschlager HHA (2005). Towards the neural basis of magnetoreception: a neuroanatomical approach. Naturwissenschaften.

[CR54] Niessner C, Denzau S, Gross JC, Peichl L, Bischof HJ, Fleissner G, Wiltschko W, Wiltschko R (2011). Avian ultraviolet/violet cones identified as probable magnetoreceptors. PLoS One.

[CR55] Olcese J, Hurlbut E (1989). Comparative studies on the retinal dopamine response to altered magnetic-fields in rodents. Brain Res.

[CR56] Olcese J, Reuss S, Semm P (1988). Geomagnetic-field detection in rodents. Life Sci.

[CR57] Olcese J, Reuss S, Stehle J, Steinlechner S, Vollrath L (1988). Responses of the mammalian retina to experimental alteration of the ambient magnetic-field. Brain Res.

[CR58] Orgel AR, Smith JC (1954). Test of the magnetic theory of homing. Science.

[CR59] Partch CL, Sancar A (2005). Photochemistry and photobiology of cryptochrome blue-light photopigments: The search for a photocycle. Photochem Photobiol.

[CR60] Pettigrew J, Wallman J, Wildsoet C (1990). Saccadic oscillations facilitate ocular perfusion from the avian pecten. Nature.

[CR61] Phillips JB (1996). Magnetic navigation. J Theor Biol.

[CR62] Phillips JB, Borland SC (1992). Behavioral evidence for use of a light-dependent magnetoreception mechanism by a vertebrate. Nature.

[CR63] Phillips JB, Borland SC (1992). Magnetic compass orientation is eliminated under near-infrared light in the eastern red-spotted newt notophthalmus-viridescens. Anim Behav.

[CR64] Phillips JB, Borland SC (1994). Use of a specialized magnetoreception system for homing by the eastern red-spotted newt notophthalmus-viridescens. J Exp Biol.

[CR65] Phillips JB, Sayeed O (1993). Wavelength-dependent effects of light on magnetic compass orientation in *Drosophila*-*melanogaster*. J Comp Physiol A.

[CR66] Phillips JB, Muheim R, Jorge PE (2010). A behavioral perspective on the biophysics of the light-dependent magnetic compass: a link between directional and spatial perception?. J Exp Biol.

[CR67] Picazo ML, Catala MD, Bardasano JL (1993). Histopathology of the harderian-gland of rodents exposed to elf magnetic-fields. Bioelectrochem Bioenerg.

[CR68] Ramirez E (2011) Is there a photo-dependent magneto-reception mechanism in the pigeon’s optic tectum? Master thesis, University of Chile (Chile)

[CR69] Ritz T, Adem S, Schulten K (2000). A model for photoreceptor-based magnetoreception in birds. Biophys J.

[CR70] Ritz T, Dommer DH, Phillips JB (2002). Shedding light on vertebrate magnetoreception. Neuron.

[CR71] Rodgers CT, Hore PJ (2009). Chemical magnetoreception in birds: the radical pair mechanism. PNAS.

[CR72] Rose J (2005) The neural basis of avian magnetic orientation. Master thesis, University of Otago (New Zeland)

[CR73] Rowe C (1999). Receiver psychology and the evolution of multicomponent signals. Anim Behav.

[CR74] Schneider T (1995). Distribution of 2-[i-125]iodomelatonin binding-sites in the brain of the pied flycatcher (*Ficedula*-*hypoleuca*) and the zebra finch (*Taeniopygia*-*guttata*). J Exp Biol.

[CR75] Schulten K, Swenberg C, Weller A (1978). A biomagnetic sensory mechanism based on magnetic field modulated coherent electron spin motion. Z Phys Chem.

[CR76] Semm P (1983). Neurobiological investigations on the magnetic sensitivity of the pineal gland in rodents and pigeons. Comp Biochem Physiol A.

[CR77] Semm P, Beason RC (1990). Responses to small magnetic variations by the trigeminal system of the bobolink. Brain Res Bull.

[CR78] Semm P, Beason RC (1990). Sensory basis of bird orientation. Experientia.

[CR79] Semm P, Demaine C (1986). Neurophysiological properties of magnetic cells in the pigeon’s visual system. J Comp Physiol A.

[CR80] Semm P, Nohr D, Demaine C, Wiltschko W (1984). Neural basis of the magnetic compass interactions of visual magnetic and vestibular inputs in the pigeon’s brain. J Comp Physiol A.

[CR81] Shcherbakov VP, Winklhofer M (1999). The osmotic magnetometer: a new model for magnetite-based magnetoreceptors in animals. Eur Biophys J.

[CR82] Solov´yov I, Domratcheva T, Schulten K. (2014) Separation of photo-induced radical pair in cryptochrome to a functionally critical distance. Sci Rep 4:3845. doi:10.1038/srep03845 10.1038/srep03845 PMC4894384 24457842

[CR83] Stehle J, Reuss S, Schroder H, Henschel M, Vollrath L (1988). Magnetic-field effects on pineal *n*-acetyltransferase activity and melatonin content in the gerbil—role of pigmentation and sex. Physiol Behav.

[CR84] Taube JS (1998). Head direction cells and the neurophysiological basis for a sense of direction. Prog Neurobiol.

[CR85] Thoss F, Bartsch B (2003). The human visual threshold depends on direction and strength of a weak magnetic field. J Comp Physiol A.

[CR86] Thoss F, Bartsch B, Fritzsche B, Tellschaft D, Thoss M (2000). The magnetic field sensitivity of the human visual system shows resonance and compass characteristic. J Comp Physiol A.

[CR87] Thoss F, Bartsch B, Tellschaft D, Thoss M (2002). The light sensitivity of the human visual system depends on the direction of view. J Comp Physiol A.

[CR88] Tian L, Xiao B, Lin W, Zhang S, Zhu R, Pan Y (2007). Testing for the presence of magnetite in the upper-beak skin of homing pigeons. Biometals.

[CR89] Treiber CD, Salzer MC, Riegler J, Edelman N, Sugar C, Breuss M, Pichler P, Cadiou H, Saunders M, Lythgoe M, Shaw J, Keays DA (2012). Clusters of iron-rich cells in the upper beak of pigeons are macrophages not magnetosensitive neurons. Nature.

[CR90] Vargas JP, Siegel JJ, Bingman VP (2006). The effects of a changing ambient magnetic field on single-unit activity in the homing pigeon hippocampus. Brain Res Bull.

[CR91] Walcott C, Green RP (1974). Orientation of homing pigeons altered by a change in the direction of the applied magnetic field. Science.

[CR92] Walcott C, Gould JL, Lednor AJ (1988). Homing of magnetized and demagnetized pigeons. J Exp Biol.

[CR93] Wallman J, Letelier JC (1993). Eye movements, head movements and gaze stabilization in birds. Vision, Brain and Behaviour in birds.

[CR94] Wallraff HG, Sinsch U (1988). The role of outward-journey information in homing experiments with pigeons—new data on ontogeny of navigation and general survey. Ethology.

[CR95] Wiltschko W (1968). Uber den einfluss statischer magnetfelder auf die zugorientierung der rotkehlchen (*Erithacus rubecula*). Z Tierpsychol.

[CR96] Wiltschko W, Wiltschko R (1995). Migratory orientation of european robins is affected by the wavelength of light as well as by a magnetic pulse. J Comp Physiol A.

[CR97] Wiltschko W, Wiltschko R (2001). Light-dependent magnetoreception in birds: the behaviour of european robins, *Erithacus rubecula*, under monochromatic light of various wavelengths and intensities. J Exp Biol.

[CR98] Wiltschko W, Wiltschko R (2002). Magnetic compass orientation in birds and its physiological basis. Naturwissenschaften.

[CR99] Wiltschko W, Wiltschko R (2005). Magnetic orientation and magnetoreception in birds and other animals. J Comp Physiol A.

[CR100] Wiltschko R, Wiltschko W (2006). Magnetoreception. Bioessays.

[CR101] Wiltschko W, Wiltschko R (2007). Magnetoreception in birds: two receptors for two different tasks. J Ornithol.

[CR102] Wiltschko W, Munro U, Ford H, Wiltschko R (1993). Red-light disrupts magnetic orientation of migratory birds. Nature.

[CR103] Wiltschko W, Munro U, Beason RC, Ford H, Wiltschko R (1994). A magnetic pulse leads to a temporary deflection in the orientation of migratory birds. Experientia.

[CR104] Wiltschko W, Munro U, Ford H, Wiltschko R (2003). Lateralisation of magnetic compass orientation in silvereyes, *Zosterops lateralis*. Aust J Zool.

[CR105] Wiltschko W, Moller A, Gesson M, Noll C, Wiltschko R (2004). Light-dependent magnetoreception in birds: analysis of the behaviour under red light after pre-exposure to red light. J Exp Biol.

[CR106] Wiltschko W, Munro U, Ford H, Wiltschko R (2006). Bird navigation: what type of information does the magnetite-based receptor provide?. Proc R Soc Lond Biol.

[CR107] Wiltschko R, Munro U, Ford H, Stapput K, Wiltschko W (2008). Light-dependent magnetoreception: orientation behaviour of migratory birds under dim red light. J Exp Biol.

[CR108] Wiltschko R, Stapput K, Thalau P, Wiltschko W (2010). Directional orientation of birds by the magnetic field under different light conditions. J R Soc Interface.

[CR109] Wiltschko R, Dehe L, Gehring D, Thalau P, Wiltschko W (2013). Interaction between the visual and the magnetoreception system: different effects of bichromatic light regimes on the directional behavior of migratory birds. J Physiol (Paris).

[CR110] Winklhofer M (2010). Magnetoreception. J R Soc Interface.

[CR111] Winklhofer M, Kirschvink JL (2010). A quantitative assessment of torque-transducer models for magnetoreception. J R Soc Interface.

[CR112] Wu L-Q, Dickman JD (2011). Magnetoreception in an avian brain in part mediated by inner ear lagena. Curr Biol.

[CR113] Wu L-Q, Dickman JD (2012). Reports neural correlates of a magnetic sense. Science.

[CR114] Yano A, Sato A, Miyata T, Mizutani Y, Sakaki Y, Kitamura S, Ikuta K, Ogura M (1996). Behavioral tests for magnetic sensitivity of hime salmon (kokanee: Land-locked sockeye salmon *Oncorhynchus nerka*). Nippon Suisan Gakkaishi.

[CR115] Yeagley HL, Whitmore FC (1947). A preliminary study of a physical basis of bird navigation. J Appl Phys.

[CR116] Zapka M, Heyers D, Hein CM, Engels S, Schneider N-L, Hans J, Weiler S, Dreyer D, Kishkinev D, Wild JM, Mouritsen H (2009). Visual but not trigeminal mediation of magnetic compass information in a migratory bird. Nature.

[CR117] Zapka M, Heyers D, Liedvogel M, Jarvis ED, Mouritsen H (2010). Night-time neuronal activation of cluster N in a day- and night-migrating songbird. Eur J Neurosci.

